# Precision of attenuation coefficient measurements by optical coherence tomography

**DOI:** 10.1117/1.JBO.27.8.085001

**Published:** 2022-08-09

**Authors:** Linda B. Neubrand, Ton G. van Leeuwen, Dirk J. Faber

**Affiliations:** a Amsterdam UMC, Location AMC, University of Amsterdam, Department of Biomedical Engineering and Physics, Amsterdam, The Netherlands; b Amsterdam Cardiovascular Sciences, Atherosclerosis and Ischemic Syndromes, Amsterdam, The Netherlands; c Cancer Center Amsterdam, Imaging and Biomarkers, Amsterdam, The Netherlands

**Keywords:** optical coherence tomography, attenuation coefficient, Cramér–Rao lower bound, maximum likelihood estimation, curve-fitting, Fisher-information matrix, OCT signal simulation

## Abstract

**Significance:**

Optical coherence tomography (OCT) is an interferometric imaging modality, which provides tomographic information on the microscopic scale. Furthermore, OCT signal analysis facilitates quantification of tissue optical properties (e.g., the attenuation coefficient), which provides information regarding the structure and organization of tissue. However, a rigorous and standardized measure of the precision of the OCT-derived optical properties, to date, is missing.

**Aim:**

We present a robust theoretical framework, which provides the Cramér –Rao lower bound $\sigma_{\mu_{\mathrm{OCT}}}$ for the precision of OCT-derived optical attenuation coefficients.

**Approach:**

Using a maximum likelihood approach and Fisher information, we derive an analytical solution for $\sigma_{\mu_{\mathrm{OCT}}}$ when the position and depth of focus are known. We validate this solution, using simulated OCT signals, for which attenuation coefficients are extracted using a least-squares fitting procedure.

**Results:**

Our analytical solution is in perfect agreement with simulated data without shot noise. When shot noise is present, we show that the analytical solution still holds for signal-to-noise ratios (SNRs) in the fitting window being above 20 dB. For other cases (SNR<20  dB, focus position not precisely known), we show that the numerical calculation of the precision agrees with the $\sigma_{\mu_{\mathrm{OCT}}}$ derived from simulated signals.

**Conclusions:**

Our analytical solution provides a fast, rigorous, and easy-to-use measure for OCT-derived attenuation coefficients for signals above 20 dB. The effect of uncertainties in the focal point position on the precision in the attenuation coefficient, the second assumption underlying our analytical solution, is also investigated by numerical calculation of the lower bounds. This method can be straightforwardly extended to uncertainty in other system parameters.

## 1 Introduction

Optical coherence tomography (OCT) is an imaging technique based on low-coherence interferometry, providing cross-sectional views into the subsurface structure of samples. OCT creates depth resolved images with a high-spatial resolution, commonly in the range of 5 to 15  μm. OCT is widely used in numerous disciplines, ranging from diagnostic medicine to art conservation.

Next to visualization, OCT also provides additional information about physiological properties such as blood content, as well as tissue structure and organization.^1^^,^^2^ This information is extracted from the optical properties^3^^,^^4^ of the investigated tissue and therefore the clinical value of this information will depend strongly on the precision with which these properties can be determined. One important optical property is the so-called attenuation coefficient $\mu_{\mathrm{OCT}}\;({\mathrm{mm}}^{-1})$, which is a measure of the decay of light intensity within the sample due to absorption and scattering. Changes of absorption and scattering properties of the investigated tissue are thus reflected in the attenuation coefficient and can be used for tissue characterization, such as in cancer detection.^2^^,^^5^^,^^6^ Promising results have been shown in medical fields, including cardiology, dermatology, and urology.^7^^–^^14^ To distinguish different structures and to extract reliable optical information from tissue, it is crucial that the $\mu_{\mathrm{OCT}}$ is measured accurately and precisely. The main cause of imprecision is speckle. OCT speckle is the voxel-to-voxel variation of OCT amplitude, due to random variations in the spatial position of scattering particles within the imaging voxel. Randomly placed scatterers within the voxels will thus return scattered fields with random amplitude and phase, leading to intensity fluctuations at the detector. Another cause of fluctuation is (shot) noise. These fluctuations ultimately limit the precision with which the attenuation coefficient can be obtained.

The common approach to extract the attenuation coefficient is to select an axial fitting range (AFR) and apply a nonlinear least squares (NLLS) curve fit using an appropriate signal model.^15^ This model features the optical properties of interest as well as parameters that characterize the OCT system itself. For maximum likelihood estimation (MLE) methods, the maximum obtainable precision of the parameters can be calculated using a Cramér–Rao analysis^16^ based on the Fisher-information matrix (FIM) $F_{\theta }$. The latter is a measure of the amount of information about the parameter set θ that is present in the observed data. The inverse of the FIM $F_{\theta }^{-1}$ represents a covariance matrix, in which the diagonal elements represent the variance of the parameters and the off-diagonal elements represent the covariance between parameters. The Cramér–Rao inequality reads $\mathrm{cov}(\theta ,\theta )\geq F_{\theta }^{-1}$ and asserts that no unbiased estimator can be found for which the variance of the estimated parameters is lower than the diagonal elements of the covariance matrix $F_{\theta }^{-1}$.^16^ For observed data that are normally distributed, NLLS curve fitting ($\chi^{2}$-minimization) is a form of unbiased MLE and thus should be capable of reaching the Cramér–Rao lower bound (CRLB) to achieve the maximum attainable precision. For ease of interpretation, the CRLB will be expressed here as a standard deviation (square root of the diagonal elements of $F_{\theta }^{-1}$), so it has the same physical units as the parameters it applies to [e.g., $\sigma_{\mu_{\mathrm{OCT}}}\;({\mathrm{mm}}^{-1})$]. When the MLE procedure would be repeated a large number of times on statistically independent “realizations” of the dataset, the CRLB can be interpreted as the lowest attainable value of the standard deviation of the distribution of the resulting parameter values.

Here, we present an analytical expression for the CRLB for the attenuation coefficient. It is derived from the FIM for speckled OCT data, under the assumption that noise is absent, and the focal point position, the Rayleigh length of the optical system, and the sensitivity roll-off in depth are known. The limit of validity of the expression under the first assumption is investigated numerically at different noise levels while assuming a shot noise limited system. The effect of uncertainties in the focal point position on the precision in the attenuation coefficient, the second assumption underlying our analytical solution, is also investigated and can be straightforwardly extended to uncertainty in other system parameters.

## 2 Theoretical Background

Several preclinical and phantom studies have shown that the OCT signal versus depth z is best described by a single exponential decay curve, corrected with the confocal point spread function and sensitivity roll-off in depth. The resulting expression for the mean-squared OCT signal as a function of depth in the sample is^17^

$${\left\langle i_{d}(z)\right\rangle }^{2}=\alpha \cdot T(z-z_{f})\cdot H(z-z_{0})\cdot \exp(-2\mu_{\mathrm{OCT}}z)+{\left\langle \zeta \right\rangle }^{2},$$ (1)

where $\alpha =\eta \cdot \mu_{b,\mathrm{NA}}$ is a conversion factor that includes the detector response; $\mu_{b,\mathrm{NA}}$ is the backscattering coefficient within the numerical aperture (NA) of the detection system; ${\left\langle \zeta \right\rangle }^{2}$ is the mean-squared noise floor, which is assumed to be independent of depth, and T(z) is the confocal point spread function:^18^

$$T(z-z_{f})=\frac{1}{{\left(\frac{z-z_{f}}{2nz_{R}}\right)}^{2}+1}.$$ (2)

Here, $z_{f}$ is the focus position and $2nz_{R}$ is the depth of focus, with n denoting the average refractive index along the beam and $z_{R}$ is the Rayleigh length measured in air. The function $H(z-z_{0})$ describes the sensitivity roll-off in depth for non-time domain OCT systems as^19^

$$H(z-z_{0})={\mathrm{sinc}}^{2}[\frac{\pi (z-z_{0})}{2z_{D}}]\exp\{-\frac{r^{2}}{2\;\ln\;2}{\left[\frac{\pi (z-z_{0})}{2z_{D}}\right]}^{2}\},$$ (3)

where $z_{0}$ is the distance between zero-delay and the tissue boundary; r is the ratio of optical resolution to sampling resolution; and $z_{D}=\lambda^{2}/4n\delta \lambda$ is the maximum imaging depth achievable with a spectral sampling pitch δλ and a central wavelength of λ.

In contrast to the confocal parameters, the roll-off is nowadays almost negligible for swept source-based OCT systems. To simplify the following analysis, we assume that the sensitivity roll-off can be accurately calibrated and that the signal is preprocessed to account for its influence. To include the roll-off into the theoretical framework, the following analysis can straightforwardly be extended by including the parameters r, $z_{0}$, and $z_{D}$ in the parameter set and the function H(z) in the signal model as described in Eq. (1). We proceed with the mean-squared measured signal

$${\left\langle A(z)\right\rangle }^{2}=\alpha \cdot T(z-z_{f})\cdot \exp(-2\mu_{\mathrm{OCT}}z)+{\left\langle \zeta \right\rangle }^{2}=\langle \overset{˜}{A}(z{)\rangle }^{2}+{\left\langle \zeta \right\rangle }^{2},$$ (4)

where ${\left\langle \overset{˜}{A}(z)\right\rangle }^{2}$ represents the mean-squared amplitude in the absence of noise.

In contrast to the mean-squared amplitude of Eq. (4), a single A-scan $A_{i}(z)$ is a fluctuating (random) signal due to the presence of speckle and noise. Under the assumptions of fully developed speckle and shot noise limited detection, the random amplitude at each depth follows a Rayleigh distribution.^17^^,^^20^ The amplitude variance $\sigma_{A}^{2}(z)$ is the sum of the variances due to speckle and shot noise, which are proportional to the backscattered power from the sample and the reference arm power, respectively. Note that for a Rayleigh distribution, the relation between its variance and mean is fixed as $\sigma_{A}^{2}=(\frac{c_{R}}{4}){\left\langle A(z)\right\rangle }^{2}$, where the constant $c_{R}=4(4-\pi )/\pi$ is introduced for later convenience. This relation yields to the familiar “speckle contrast” for fully developed speckle, the ratio of standard deviation, and mean amplitude, $\frac{\sigma_{A}}{{\left\langle \overset{˜}{A}(z)\right\rangle }^{2}}=\frac{\sqrt{c_{R}}}{2}=0.5227$.^21^^,^^22^

Consequently, the amplitude variance at each depth using Eq. (4) can be expressed as

$$\sigma_{A}^{2}(z)=\frac{c_{R}}{4}({\left\langle \overset{˜}{A}(z)\right\rangle }^{2}+{\left\langle \zeta \right\rangle }^{2}).$$ (5)

To reduce the amplitude fluctuations, A-scan averaging of statistically independent individual A-scans can be performed.^22^ The averaged amplitude $S(z)=\frac{1}{N}\sum_{j=1}^{N}A_{j}(z)$ remains a fluctuating quantity with mean ⟨S(z)⟩=⟨A(z)⟩ and variance $\sigma_{S}^{2}(z)=\sigma_{A}^{2}(z)/N$. This averaged amplitude, in general, follows an unspecified distribution $\rho_{S}(S)$ different from the Rayleigh distribution. However, with a sufficient number of averages N≳30, the averaged amplitudes at each depth can be adequately characterized by a normal distribution $\rho_{S}(S)=N(\langle A(z)\rangle ,\sigma_{A}^{2}/N)$,^23^ which we will assume henceforth.

**Fig. 1:**
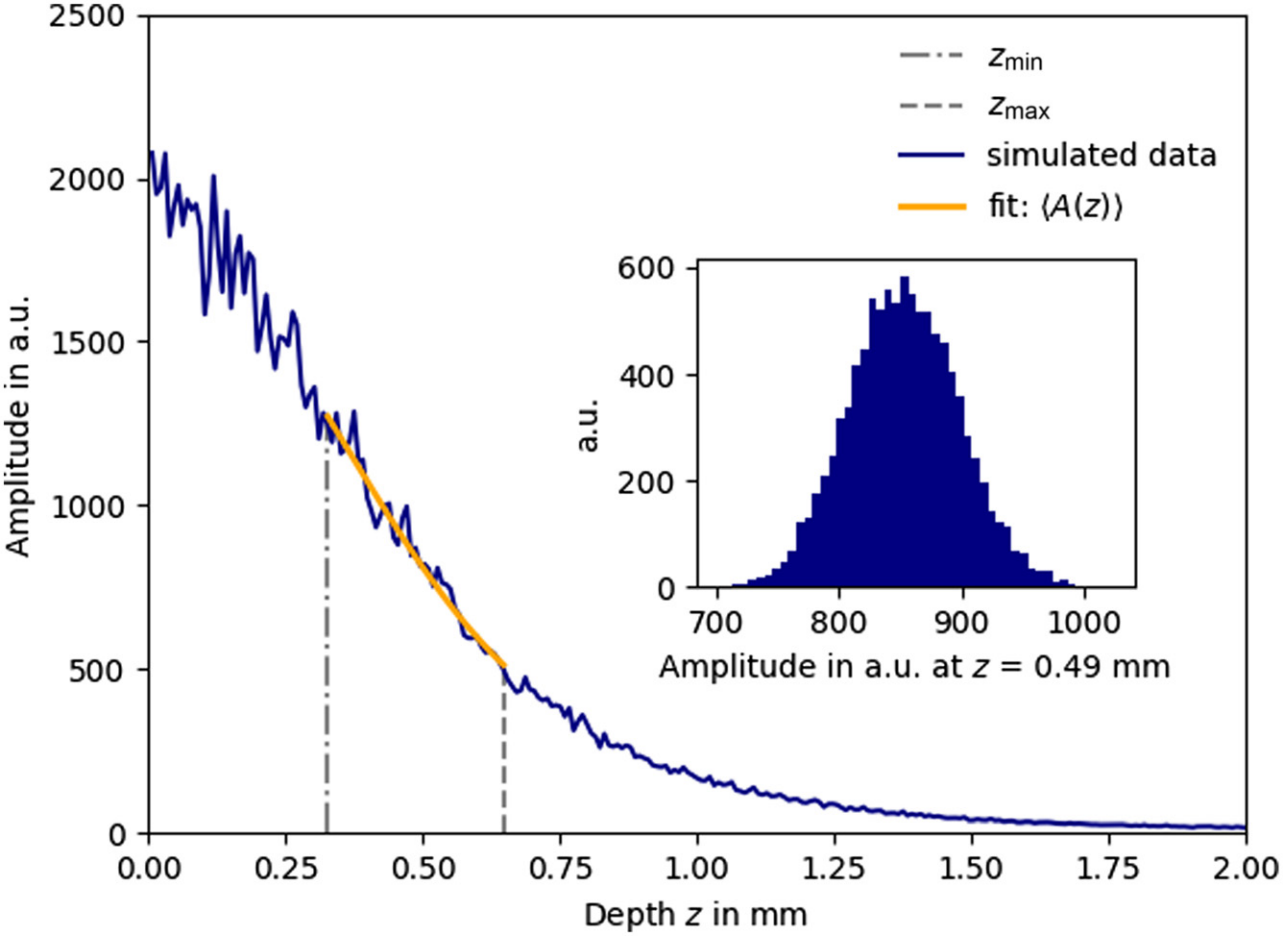
Average of N=100 independent A-scans (blue line). The amplitudes in the underlying single A-scans follow a Rayleigh distribution and are simulated using Eq. (11) using a parameter set $\theta =[\alpha =2500,\mu_{\mathrm{OCT}}=2\;{\mathrm{mm}}^{-1},z_{f}=0.3\;\mathrm{mm},\;z_{R}=0.2\;\mathrm{mm},\;\mathrm{and}\;\sigma_{\mathrm{shot}}=7$]. The two gray dashed lines indicate the boundaries of the AFR (AFR length: 328  μm and sample points: M=41). The inset depicts the normally distributed amplitude values S(z) of $10^{4}$ averaged A-scans at depth position z=0.49  mm. The amplitude at the end of the AFR is used to calculate the window-specific SNR with Eq. (13) as ${\mathrm{SNR}}_{\mathrm{AFR}}=37\;\mathrm{dB}$. The orange line depicts a curve fit using the square root of Eq. (4) as fit model with α and $\mu_{\mathrm{OCT}}$ free running.

The single A-scans, and therefore the averaged OCT signal, are fully parameterized by the parameter array $\theta =[\alpha ,\mu_{\mathrm{OCT}},z_{f},z_{R},\zeta ]$. In this parameter array, only α and $\mu_{\mathrm{OCT}}$ represent the optical properties of interest, all other parameters characterize the OCT system and can in principle be calibrated to some finite precision. The attenuation coefficient is commonly extracted trough NLLS $\chi^{2}$-fitting using the square root of Eq. (4) as fit model, which is applied on an AFR from positions ${AFR}_{\min}$ to ${AFR}_{\max}$. An example of such fit is shown in Fig. 1 (orange line).

Fluctuations in amplitude due to speckle and shot noise lead to uncertainty in the parameter estimation. To determine the smallest achievable uncertainty, i.e., the CRLB corresponding to the most precise $\mu_{\mathrm{OCT}}$ estimation, we start with the FIM matrix for the averaged A-scans^16^

$$(F_{\theta }{)}_{kl}=\overset{M}{\underset{i=1}{\sum }}E_{S}[\frac{\partial^{2}\;\log\;\rho_{S}(S(z_{i});\theta )}{\partial \theta_{k}\partial \theta_{l}}],$$ (6)

where $\rho_{S}(S)$ is the probability density function for the averaged OCT amplitude, the sum i=1…M runs over all independent averaged amplitude values within the AFR and k and l index the parameter array. The expectation value $E_{S}[\frac{\partial^{2}\;\log\;\rho_{S}(S(z_{i});\theta )}{\partial \theta_{k}\partial \theta_{l}}]=\int_{0}^{\infty }\rho_{S}(S(z_{i});\theta )[\frac{\partial^{2}\;\log\;\rho_{S}(S(z_{i});\theta )}{\partial \theta_{k}\partial \theta_{l}}]dS(z_{i})$ is calculated by integration over the probability density function $\rho_{S}(S)$. For normally distributed values of $S(z_{i})$, the FIM evaluates to^12^

$$(F_{\theta }{)}_{kl}=\overset{M}{\underset{i=1}{\sum }}{\frac{1}{\sigma_{S}^{2}(z_{i})}(\frac{\partial \langle S(z_{i})\rangle }{\partial \theta_{k}}\frac{\partial \langle S(z_{i})\rangle }{\partial \theta_{l}})|}_{\theta =\theta_{0}},$$ (7)

for M independent measurement points within the AFR and evaluated at a given set of parameters $\theta_{0}$. Here, $\left\langle S(z_{i})\right\rangle$ is the expectation value of the averaged OCT amplitude at depth $z_{i}$, and $\sigma_{S}^{2}(z_{i})$ is the corresponding variance. In terms of OCT amplitude mean and variance,

$${\left(F_{\theta }\right)}_{kl}=\overset{M}{\underset{i=1}{\sum }}\frac{N}{\sigma_{A}^{2}(z_{i})}{\left(\frac{\partial \left\langle A(z_{i})\right\rangle }{\partial \theta_{k}}\frac{\partial \langle A(z_{i})\rangle }{\partial \theta_{l}})\right|}_{\theta =\theta_{0}},$$ (8)

where N is the number of averages in the averaged A-scans, ⟨A(z)⟩ is obtained from the square root of Eq. (4), and $\sigma_{A}^{2}(z_{i})$ is obtained from Eq. (5).

$F_{\theta }$ quantifies the variation in signal likelihood caused by variations in the parameters of the signal model. By inversion of the FIM, we obtain the variation in estimated model parameters due to variation in the signal likelihood. This inverse of the FIM, $F_{\theta }^{-1}$, constitutes a quadratic covariance matrix, in which the diagonal elements represent the variances for each parameter in the set $\theta =[\alpha ,\mu_{\mathrm{OCT}},z_{f},z_{R},\zeta ]$ (and the off-diagonal elements represent covariances). We express the CRLB on the precision as a standard deviation, e.g., as the square root of the diagonal element values of $F_{\theta }^{-1}$. In our case, the CRLB of the precision of the attenuation coefficient ($\sigma_{\mu_{\mathrm{OCT}}}$) is then given by the square root of the second diagonal element of $F_{\theta }^{-1}$. When the fitting procedure would be repeated a large number of times on statistically independent “realizations” of the dataset, the CRLB can be interpreted as the lowest attainable value of the standard deviation of the distribution of the resulting attenuation coefficient values.

For the present set of parameters, Fisher-information and covariance matrices of dimensions 5×5 are obtained.

In contrast, by assuming $z_{f}$ and $z_{R}$ as exactly known and shot noise as being negligible and thus absent from the model, the parameter array is $\theta =[\alpha ,\mu_{\mathrm{OCT}}]$ and the FIM becomes a 2×2 matrix whose inversion can be computed analytically. Approximating the summation in Eq. (8) as an integration, a closed-form expression for the CRLB for the attenuation coefficient $\sigma_{\mu_{\mathrm{OCT}}}$ can be obtained (Appendix A):

$$\sigma_{\mu_{\mathrm{OCT}}}=\frac{1}{{AFR}_{\max}-{AFR}_{\min}}\sqrt{\frac{3\cdot c_{R}}{M\cdot N}}.$$ (9)

Equation (9), the main theoretical result of this article, shows that under the present assumptions, the maximum precision obtainable for $\mu_{\mathrm{OCT}}$ depends only on the axial size of the AFR, the number of independent points (M) in the AFR, and the number of independent preaverages (N). The $\sigma_{\mu_{\mathrm{OCT}}}$ is independent of the attenuation coefficient and the amplitude values. When noise is nonnegligible, it is not possible to find an analytical solution, and the lower bound has to be calculated numerically.

In practice, the focal point position in tissue $z_{f}$ will not be known exactly but with some *a priori* determined or estimated precision $p_{z_{f}}$, expressed in the same units as $z_{f}$. Calculation of the CRLB using Eq. (9), based on a 2×2 FIM in which the focal position is assumed to be exactly known ($p_{z_{f}}=0$), will yield an overestimation of the best attainable precision in $\mu_{\mathrm{OCT}}$. However, the calculation of the CRLB based on a 3×3 FIM, with $\theta =[\alpha ,\mu_{\mathrm{OCT}},z_{f}]$ may yield an underestimation of the best attainable precision because in that case $z_{f}$ is a free running parameter in the fit, allowed to take all possible values ($p_{z_{f}}=\infty$). Some variation of $\mu_{\mathrm{OCT}}$ can then be compensated by variation in $z_{f}$. Following the procedure in Ref. 24, it is possible to compute the change in $\sigma_{\mu_{\mathrm{OCT}}}$ by including prior knowledge of the precision in $z_{f}$ as follows:

$$\sigma_{\mu_{\mathrm{OCT}}}={\overset{˜}{\sigma }}_{\mu_{\mathrm{OCT}}}\sqrt{1-P_{\left(\mu_{\mathrm{OCT}},z_{f}\right)}^{2}\frac{{\overset{˜}{\sigma }}_{z_{f}}^{2}}{p_{z_{f}}^{2}+{\overset{˜}{\sigma }}_{z_{f}}^{2}}},$$ (10)

where ${\tilde{\sigma }}_{\mu_{\mathrm{OCT}}}$ and ${\tilde{\sigma }}_{z_{f}}$ are the CRLBs assuming no prior information on both parameters, $P_{\left(\mu_{\mathrm{OCT}},z_{f}\right)}=\sigma_{\left(\mu_{\mathrm{OCT}},z_{f}\right)}^{2}/\sqrt{{\overset{˜}{\sigma }}_{\mu_{\mathrm{OCT}}}^{2}{\overset{˜}{\sigma }}_{z_{f}}^{2}}$ is the normalized covariance between the attenuation coefficient, and focal point position $\left(P_{\left(\mu_{\mathrm{OCT}},z_{f}\right)}\leq 1\right)$. With increasing precision of the focal point position due to calibration (e.g., $p_{z_{f}}\to 0$), the CRLB ${\tilde{\sigma }}_{\mu_{\mathrm{OCT}}}$ decreases. Note that the extent of improvement depends on the magnitude of $P_{\left(\mu_{\mathrm{OCT}},z_{f}\right)}$: for very weak covariance between both parameters, the improvement in the CRLB will be small. In this case too, an analytical expression cannot be found and the CRLB must be calculated numerically.

## 3 Methods

We performed three sets of numerical experiments, all implemented in Python 3.8.2. For each, N single A-scans are simulated using Eq. (11) with the parameters set as α=2500, $\mu_{\mathrm{OCT}}=1,2,5,10\;{\mathrm{mm}}^{-1}$, $z_{f}=0.3\;\mathrm{mm}$, and $z_{R}=0.2\;\mathrm{mm}$. Each A-scan consisted of 250 independent data points, over a simulated range of 2 mm (δz=8  μm increments). These settings correspond to typical system parameters for nonophthalmic OCT systems. To simulate A-scans with Rayleigh distributed amplitudes, we used the standard method of sampling^25^ based on inversion of the continuous distribution function:

$$A_{\mathrm{sim}}(z_{i})=\sqrt{-\frac{4}{4-\pi }\cdot \sigma_{A}^{2}(z_{i})\cdot \ln(\xi_{\left[0,1\right]})},$$ (11)

where $\xi_{\left[0,1\right]}$ is a uniformly distributed random number between 0 and 1. The total variance $\sigma_{A}^{2}(z_{i})$ is calculated according to Eq. (5). These A-scans are subsequently laterally averaged, $S_{\mathrm{sim}}(z_{i})=\frac{1}{N}\overset{N}{\underset{j=1}{\sum }}A_{\mathrm{sim},j}(z_{i})$. Next to the mean, for each data point the variance $\sigma_{S_{\mathrm{sim}}}^{2}(z_{i})$ is also computed. Values on the discrete depth axis are given by $z_{i}=(i-\frac{1}{2})$ δz with i=1…N.

The attenuation coefficient is found by a Levenberg–Marquardt NLLS curve-fitting procedure applied to the averaged A-scan, with either two ($\alpha ,\mu_{\mathrm{OCT}}$) or three ($\alpha ,\mu_{\mathrm{OCT}},z_{f}$) or all parameters except the noise floor $\left(\alpha ,\mu_{\mathrm{OCT}},z_{f},z_{R}\right)$ free running, as specified below, and the other parameters fixed at the input value of the simulations. This procedure finds the model parameters by minimizing the variance-weighted sum of squared residuals $\chi^{2}$ in the AFR according to

$$\chi^{2}=\frac{\sum_{i=1}^{M}{\left[S_{\mathrm{sim}}(z_{i})-A(z_{i})\right]}^{2}}{\sigma_{S_{\mathrm{sim}}}^{2}(z_{i})},$$ (12)

where the fit model $A(z_{i})$ is obtained from the square root of Eq. (4). Fit procedures are repeated $10^{4}$ times to calculate the mean and standard deviation of the distribution of $\mu_{\mathrm{OCT}}$ estimations. This obtained standard deviation is then compared to the CRLB for the specific experiment, calculated numerically based on Eq. (8)—as the square root of the second diagonal element of $F_{\theta }^{-1}$—or analytically using Eq. (9). To quantify the effect of noise on the $\mu_{\mathrm{OCT}}$ estimation, we calculate an AFR-specific signal-to-noise metric as^26^

$${\mathrm{SNR}}_{\mathrm{AFR}}=20\cdot \log_{10}(\frac{S({\mathrm{AFR}}_{\max})}{\sigma_{\text{shot}}}),$$ (13)

using the amplitude at the maximum depth of the AFR $S({\mathrm{AFR}}_{\max})$ and the standard deviation of the shot noise $\sigma_{\text{shot}}=\sqrt{\frac{c_{R}}{4}}\langle \zeta \rangle$.

In the first experiment, we compare the standard deviation of the $10^{4}$ fitted attenuation coefficients to the CRLB on the precision of $\mu_{\mathrm{OCT}}$ as a function of the number of averaged A-scans N. The CRLB is calculated numerically as well as with the analytical solution of Eq. (9), in the absence of shot noise (ζ=0, $\sigma_{\text{shot}}=0$), and numerically in the presence of shot noise (ζ=13.5, $\sigma_{\text{shot}}=7$). The shot noise value is arbitrarily chosen and corresponds to the signal-to-noise ratio (SNR) value encountered in practice. The AFR was 2 mm. Values for averaging were linearly spaced between 2 and 110.

In the second experiment, we investigate the influence of the noise level in the AFR on the precision of the attenuation coefficient estimation by shifting an |AFR|=328  μm starting at z=0 down to a depth of 6 mm to vary ${\mathrm{AFR}}_{\max}$ and thus ${\mathrm{SNR}}_{\mathrm{AFR}}$ in Eq. (13). Simulations are done using ζ=0.191 ($\sigma_{\text{shot}}=0.1$) to facilitate ${\mathrm{SNR}}_{\mathrm{AFR}}$ up to 80 dB. We lowered the noise level to ensure that the full range up to 60 dB of the SNRs is covered.

In the third experiment, we investigate the influence of prior knowledge of the precision in focal point position $z_{f}$ in the presence (ζ=13.5, $\sigma_{\text{shot}}=7$) of noise. We calculated the CRLB in the case of two free parameters with two ($\alpha ,\mu_{\mathrm{OCT}}$) or three ($\alpha ,\mu_{\mathrm{OCT}},z_{f}$) free parameters. The former case indicates complete knowledge of the focal point position, the latter case indicates no knowledge at all. Equation (10) predicts the achievable precision when some knowledge, between these two extremes, is available. To simulate this situation, we implemented a constrained NLLS fitting algorithm, in which the parameter $z_{f}$ was only allowed to vary between in a restricted range around the true value, 0.3 mm $\pm p_{z_{f}}$ where $p_{z_{f}}$ was increased from 0.001 to 0.05 mm with a step size of 0.001. The AFR was 2 mm.

## 4 Results

An example of a simulated 100 times averaged A-scan S(z) is shown in Fig. 1 (dark blue line). In this simulation, the parameter set of $\theta =[\alpha =2500,\mu_{\mathrm{OCT}}=2\;{\mathrm{mm}}^{-1},z_{f}=0.3\;\mathrm{mm},z_{R}=0.2\;\mathrm{mm},\text{ and }\sigma_{\text{shot}}=7]$ was used. The orange line depicts an NLLS fit using the square root of Eq. (4) as fit model, with α and $\mu_{\mathrm{OCT}}$ free running. The boundaries of the |AFR|=328  μm are given by the vertical gray lines. The inset shows the distribution of $10^{4}$ values of the S(z) values at position z=0.49  mm and demonstrates that the data points used in the fit can assumed as being normally distributed. The mean and standard deviation of $10^{4}$ values of the fitted parameters are α=2500±21 and $\mu_{\mathrm{OCT}}=1.999\pm 0.088\;{\mathrm{mm}}^{-1}$.

For the example given, numerical evaluation of the CRLB using Eq. (8) yields $\sigma_{\theta }=[\sigma_{\alpha }=112,\sigma_{\mu_{\mathrm{OCT}}}=0.0863\;{\mathrm{mm}}^{-1}]$. The corresponding CLRB from the noise-less analytical expression Eq. (9) is $\sigma_{\mu_{\mathrm{OCT},\mathrm{an}}}=0.0862\;{\mathrm{mm}}^{-1}$, indicating that the influence of noise is nearly negligible in this simulation. We compared the CRLB for the attenuation coefficient using different combination of fixed and free running parameters, shown in Table 1 (an extended version is shown in Table 2 in Appendix B).

**Table 1:** CRLB for precision of attenuation coefficient estimation at $\mu_{\mathrm{OCT}}=2\;{\mathrm{mm}}^{-1}$; and corresponding simulation results for different parameters fixed or free running in the fitting procedure. Parameter and simulation values are equal to those in Fig. 1. The CRLB marked with (Γ) is calculated using the analytical expression Eq. (9); other values are calculated numerically from Eq. (8). The simulation results are the standard deviation of $10^{4}$ $\mu_{\mathrm{OCT}}$ estimations.

Free running (✓) or fixed (–) parameter				$\sigma_{\mu_{\mathrm{OCT}}}\;({\mathrm{mm}}^{-1})$			
A	$\mu_{\mathrm{OCT}}$	$z_{f}$	$z_{R}$	$\sigma_{\mathrm{shot}}=0$		$\sigma_{\mathrm{shot}}=7$	
2500	$2\;{\mathrm{mm}}^{-1}$	0.3 mm	0.2 mm	CRLB	sim	CRLB	sim
✓	✓	—	—	0.086^a^	0.088	0.086	0.087
✓	✓	✓	—	0.34	0.46^b^	0.34	0.45^b^
✓	✓	—	✓	0.89	0.67^b^	0.89	0.67^b^
✓	✓	✓	✓	2.5	1.8^b^	2.5	1.7^b^

a Calculated from Eq. (9).

b Fitted $\mu_{\mathrm{OCT}}$ values were not normally distributed.

With relatively high ${\mathrm{SNR}}_{\mathrm{AFR}}=37\;\mathrm{dB}$, the influence of noise is not visible. This table clearly illustrates that precise knowledge of the confocal parameters $z_{f}$ and $z_{R}$, which removes them as variable parameters in the estimation procedure, increases the achievable precision in the attenuation coefficient. No precise determination of the attenuation coefficient is possible when all parameters are free running (bottom row). Essentially, the fit in the selected AFR is now overparameterized, and variations in $\mu_{\mathrm{OCT}}$ can be counteracted by variations in other parameters to maintain a minimum $\chi^{2}$. In the two intermediate cases, with either $z_{f}$ or $z_{R}$ free running, the CRLB is not reached using the employed NLLS curve-fitting algorithm.

**Fig. 2:**
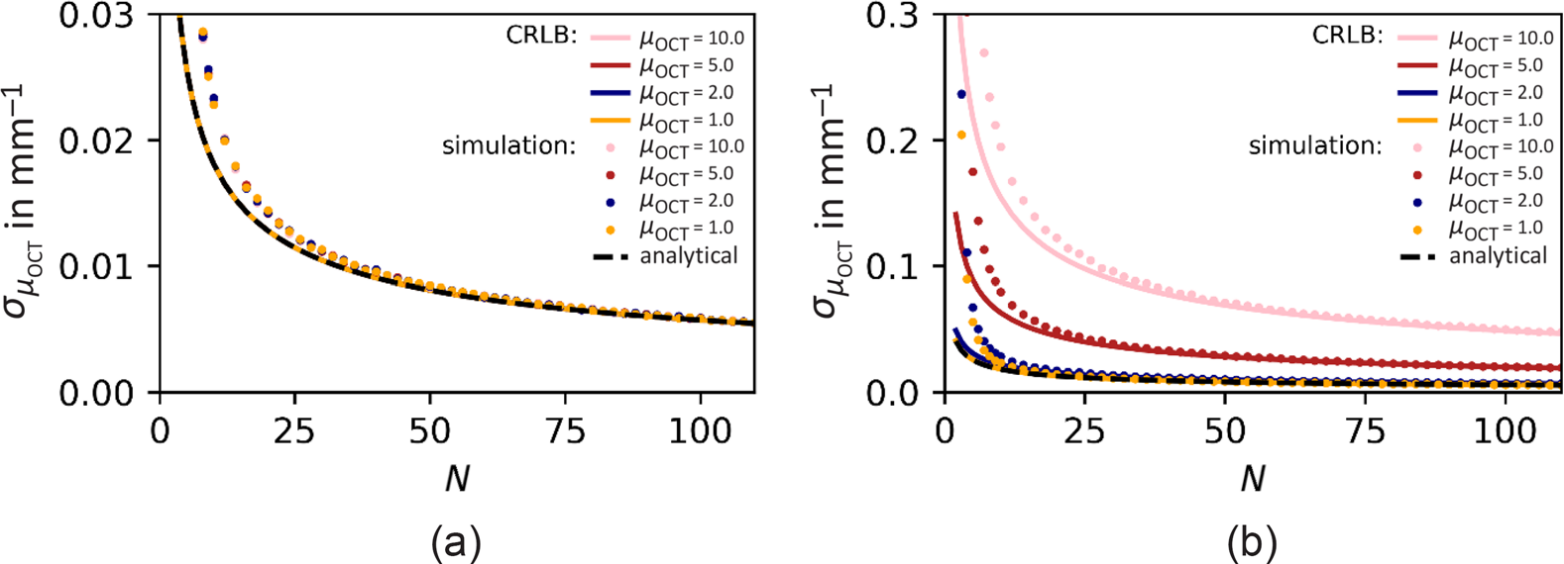
CRLB on the precision of $\mu_{\mathrm{OCT}}$ (mm^−1^) (lines) as a function of the number of averaged A-scans N in (a) the absence and (b) presence of shot noise ($\sigma_{\mathrm{shot}}=7$). The CRLB is calculated analytically using Eq. (9) (dashed lines) and numerically based on Eq. (8) (solid lines). Model parameters $\theta =[\alpha =2500,\mu_{\mathrm{OCT}}=[1,2,5,10]\;{\mathrm{mm}}^{-1},z_{f}=0.3\;\mathrm{mm},\text{ and }z_{R}=0.2\;\mathrm{mm}]$. The dots each show the standard deviation of $10^{4}$ fitted $\mu_{\mathrm{OCT}}$ values using the square root of Eq. (4) as fit model, with α and $\mu_{\mathrm{OCT}}$ free running. AFR=2  mm with M=250 data points.

The result of the first numerical experiment is shown in Fig. 2, which shows the precision of the attenuation coefficient determination as a function of the number of averages N. We used an AFR equal to the full penetration depth of 2 mm in the absence (a) and presence of shot noise (b). Please note the different vertical scales. The confocal parameters are fixed at $z_{f}=0.3\;\mathrm{mm}$, $z_{R}=0.2\;\mathrm{mm}$. The dashed black line in both panels represents the CRLB $\sigma_{\mu_{\mathrm{OCT}}}$ in the absence of noise using Eq. (9), and the colored lines indicate the calculated $\sigma_{\mu_{\mathrm{OCT}}}$ associated with the particular values of $\mu_{\mathrm{OCT}}$. The colored dots each indicate the standard deviation of $10^{4}$ $\mu_{\mathrm{OCT}}$ fits to the averaged A-line.

The predicted $1/\sqrt{N}$ dependency of the precision is visible for the analytically and numerically calculated $\sigma_{\mu_{\mathrm{OCT}}}$ and is verified by the simulation results. For small N (<30), the distribution of averaged amplitudes S(z) is not sufficiently approximated by a normal distribution, leading to a small difference between the predicted CRLB and the standard deviation of the simulated data. Figure 2(a) shows that the calculated and simulated precisions overlap perfectly if the averaged amplitudes can be assumed as being normal distributed (number of averages N≳30, see also Appendix C). In the absence of noise [Fig 2(a)], the simulation results and CRLB predictions for $\mu_{\mathrm{OCT}}=[1,2,5,10]\;{\mathrm{mm}}^{-1}$ coincide as predicted by Eq. (9). This nondependence on $\mu_{\mathrm{OCT}}$ of the precision appears lost when noise is present [Fig. 2(b)]. Better precision is predicted and obtained for lower attenuation coefficient values, all other parameters being equal. This tendency is explained by the fact that with decreasing $\mu_{\mathrm{OCT}}$, the averaged amplitude S(z) decays slower, and therefore reaches the noise level at a later point in within the fixed AFR. Consequently, with less data points falling into the noise region, the number of data points that effectively carry information on $\mu_{\mathrm{OCT}}$ is increased compared to curves with higher attenuation coefficients. For small $\mu_{\mathrm{OCT}}$, the noise-dependency remains negligible, for which reason the CRLB is described well by the analytical expression Eq. (9) in this regime.

**Fig. 3:**
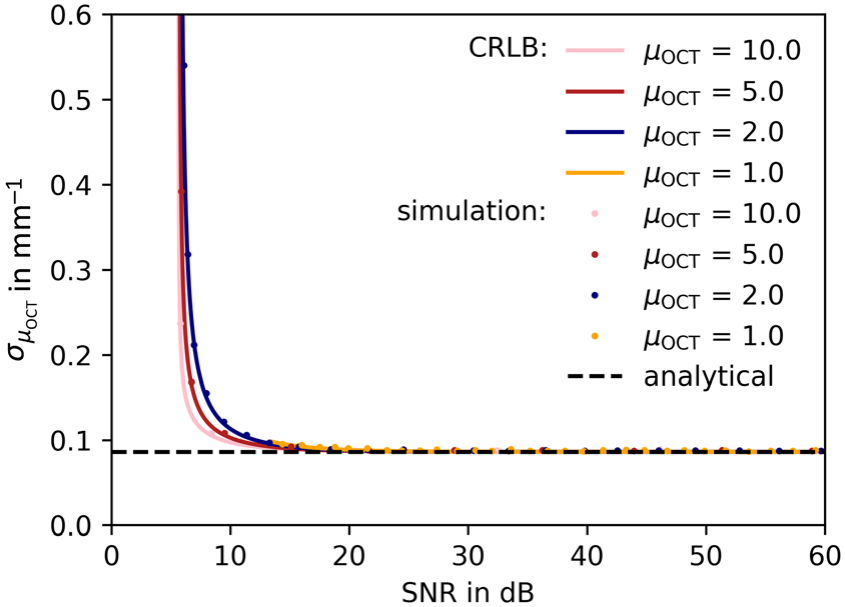
CRLB on the precision of $\mu_{\mathrm{OCT}}$ (mm^−1^) as a function of window-specific ${\mathrm{SNR}}_{\mathrm{AFR}}$. The black dashed line represents the CRLB in absence of noise, calculated with Eq. (9). Colored curves represent the CRLB based on Eq. (8). The dots each show the standard deviation of $10^{4}$ fitted $\mu_{\mathrm{OCT}}$ values. The data were simulated using $\theta =[\alpha =2500,\mu_{\mathrm{OCT}}=[1,2,5,10]\;{\mathrm{mm}}^{-1},z_{f}=0.3\;\mathrm{mm},z_{R}=0.2\;\mathrm{mm},\sigma_{\text{shot}}=0.1]$. Sliding AFR=328  μm with M=41 data points. N=100 A-scans were averaged prior to the fitting using the square root of Eq. (4) as fit model, with α and $\mu_{\mathrm{OCT}}$ free running.

To further substantiate the effect of noise in the AFR on the CRLB, in the second experiment we decreased the AFR to 328  μm (M=41 data points). By sliding the AFR in depth, the contribution of noise is increased leading to a reduced ${\mathrm{SNR}}_{\mathrm{AFR}}$ [Eq. (13)]. The reduced AFR corresponds to measurements when the $\mu_{\mathrm{OCT}}$ of a limited section of the depth scan is sought. The results are shown in Fig. 3, where the notation is kept the same as in Fig. 2.

The limiting value of $∼0.086\;{\mathrm{mm}}^{-1}$ for increasing ${\mathrm{SNR}}_{\mathrm{AFR}}$ is found from Eq. (9) and is the direct consequence of the presence of speckle. Shot noise as an additional source of signal fluctuation leads to a further decrease of achievable precision (higher $\sigma_{\mu_{\mathrm{OCT}}}$). The lowest value of ${\mathrm{SNR}}_{\mathrm{AFR}}$ in the current experiment is 5.63 dB, which is found by rewriting Eq. (13) as ${\mathrm{SNR}}_{\mathrm{AFR}}=20\cdot \log_{10}(\sqrt{\frac{4}{c_{R}}}\frac{A(z_{\max})}{\zeta })$ and setting the amplitude at the end of the AFR equal to the mean noise floor. Clearly, in this case, the data carry little information on $\mu_{\mathrm{OCT}}$ and the achievable $\sigma_{\mu_{\mathrm{OCT}}}$ grows infinitely large. For high ${\mathrm{SNR}}_{\mathrm{AFR}}$, the precision tends asymptotically toward the value obtained from the analytical expression.

Above an ${\mathrm{SNR}}_{\mathrm{AFR}}$ of 20 dB, shot noise shows negligible impact on the CRLB for the attenuation coefficient. In commonly performed OCT measurements, the SNR is usually higher within the fit range. In this SNR regime, $\sigma_{\mu_{\mathrm{OCT}}}$ is sufficiently described by Eq. (9) if $z_{f}$ and $z_{R}$ are exactly known.

In general, increasing the number of free running parameters in a fit leads to larger CRLBs, because a fit with more parameters allows more “wiggle room” for other parameters to achieve low $\chi^{2}$-values (see also the example in Table 1) especially with strong covariances between the parameters. For example, in the second and third rows in Table 1, high values for the CRLBs of both the attenuation coefficient and focus position or Rayleigh length will be computed, indicating low precision in simultaneously estimating these parameters. The more realistic intermediate approach is given by Eq. (10), where a parameter is known to some precision $p_{x}$.

**Fig. 4:**
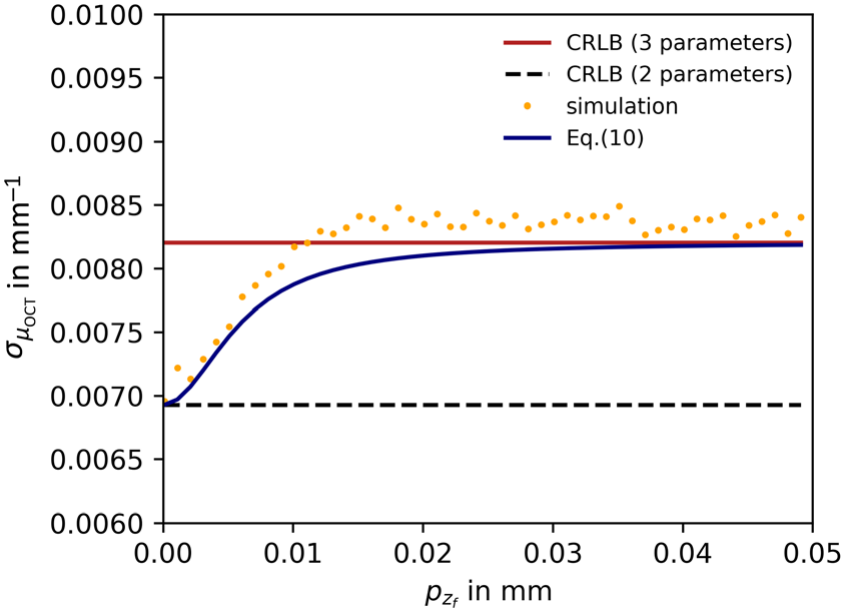
CRLB on the precision of $\mu_{\mathrm{OCT}}$ as a function of prior standard deviation of the focal point position $p_{z_{f}}$. The black dashed line represents the CRLB for a two-parameter model ($\alpha ,\mu_{\mathrm{OCT}}$), the red line represents the CRLB for a tree-parameter model ($\alpha ,\mu_{\mathrm{OCT}}$, $z_{f}$) both based on Eq. (8). The dark blue curve shows the adjusted CRLB incorporating $p_{z_{f}}$ using Eq. (10). The dots each show the standard deviation of $10^{4}$ fitted $\mu_{\mathrm{OCT}}$ values. Data are simulated using Eq. (11), $\theta =[\alpha =2500,\mu_{\mathrm{OCT}}=2\;{\mathrm{mm}}^{-1},z_{f}=0.3\;\mathrm{mm},z_{R}=0.2\;\mathrm{mm},\;\mathrm{and}\;\sigma_{\text{shot}}=7]$; AFR=2  mm with M=250 data points. N=100 A-scans were averaged prior to the constrained fitting using the square root of Eq. (4) as fit model, with α and $\mu_{\mathrm{OCT}}$ free running and $z_{f}$ allowed to vary only around $z_{f}=0.3\;\mathrm{mm}\pm p_{z_{f}}$.

We investigated the case where the focal point position is *a priori* known with some precision $p_{z_{f}}$. In the NLLS curve-fitting algorithm, this information is “incorporated” by restricting the range over which the parameter $z_{f}$ is allowed to vary to its initial value 0.3 mm $\pm p_{z_{f}}$. The result of this third experiment is shown in Fig. 4.

The black dashed line represents the CRLB $\sigma_{\mu_{\mathrm{OCT}}}=0.0069\;{\mathrm{mm}}^{-1}$, where $z_{f}$ and $z_{R}$ are assumed to be known exactly. The red line represents the CRLB ${\tilde{\sigma }}_{\mu_{\mathrm{OCT}}}=0.0082\;{\mathrm{mm}}^{-1}$ based on a 3×3-FIM assuming $z_{R}$ is known and α, $\mu_{\mathrm{OCT}}$, and $z_{f}$ are unconstrained (i.e., allowed to vary while fitting). In that latter case, the CRLB on the focal point position is ${\overset{˜}{\sigma }}_{z_{f}}=0.006\;\mathrm{mm}$. The normalized covariance between both parameters $P_{\left(\mu_{\mathrm{OCT}},z_{f}\right)}=0.54$. By including *a priori* knowledge about the precision of $z_{f}$, the CRLB ${\overset{˜}{\sigma }}_{\mu_{\mathrm{OCT}}}$ is reduced according to Eq. (10) and is shown as a blue line in Fig. 4. The yellow dots show the corresponding simulation results. Because the normalized covariance between the parameters is relatively low, it requires a prior (here $p_{z_{f}}≲0.01\;\mathrm{mm}$, which is more restrictive than the value of ${\overset{˜}{\sigma }}_{z_{f}}=0.006\;\mathrm{mm}$) before a significant improvement on $\sigma_{\mu_{\mathrm{OCT}}}$ becomes evident. The simulated values for $\mu_{\mathrm{OCT}}$ precision are slightly larger than predicted by the CRLB. A possible explanation is that the constrained nonlinear curve fitting as implemented in this experiment is not strictly an unbiased estimator.

## 5 Discussion

Quantification of the attenuation coefficient requires proper assessment of the accuracy and precision of the measurement method. This knowledge can be obtained in various ways. First, the experiments can be repeated a large number of times, after which the spread of the retrieved attenuation coefficients is assessed. When stable, fully characterized phantoms (in terms of optical properties, structure, etc.) are available, and this method will yield valuable information on accuracy and precision. Performing such series on tissue samples, however, is prone to underestimate the achievable precision because now biological variability is also factored in. Moreover, these measurements can be laborious and time consuming, and they may need to be repeated when system parameters change. Second, the measurement procedure can be numerically simulated a large number of times. This approach too can be time consuming especially when many parameters are involved, and the parameter space that must be mapped is large. We used this second approach as validation for the third method: a Cramér–Rao analysis based on the FIM, which is computationally fast and can easily be scaled to include additional parameters. The interpretation of CRLB in this article is also frequentist: when the estimation of the attenuation coefficient using an unbiased, MLE procedure would be repeated a very large number of times, and the standard deviation of the normally distributed attenuation coefficients would approach the CRLB. This concise formulation somewhat obscures the practical challenges in reaching the CRLB. First, an unbiased MLE method must be devised. For normally distributed measurement data, NLLS curve fitting is such a method, and we have ensured the normality of the measurement data by preaveraging 100 A-scans (see also Appendix C for further justification). Second, the performance of the NLLS algorithm should be such that the resulting attenuation coefficients are normally distributed. As indicated in Table 1, normally distributed values were not always the case especially when the fit model appears over parameterized or conversely, appears under parameterized, in case the model does not fit the data well. Having a biased estimation may also be the case for the data shown in Fig. 4, which was obtained using a constrained NLLS algorithm and allowed restricting fit parameter values to certain ranges.

Furthermore, the success of all three methods relies on the correctness of the fit/simulation model. In our present study, both data simulation and curve fitting were based on the same model. This assumption may be different for experimentally obtained OCT data, for example, a small fraction of multiply forward scattered light is not taken into account in the model; or when other noise sources are overlooked. Multiple scattering models are available (see the discussion in Refs. 2 and 5) and will lead to the inclusion of one more tissue parameter, e.g., the root-mean-square scattering angle or scattering anisotropy g. In our present study, we excluded the effects of sensitivity roll-off in depth for simplicity. It can be added straightforwardly at the additional cost of two parameters. Possibly, a distinction between the absorption and scattering contribution to the attenuation is desired. Then, such a comprehensive model would have the parameter array $\theta =[\alpha ,\mu_{a},\mu_{s},g,z_{f},z_{R},r,z_{0},z_{D},\zeta ]$ of which only three ($\mu_{a},\mu_{s},g$) are tissue-specific “target parameters,” and for which a high covariance between some of them can be expected. Because of this, wide CRLBs on the target parameters will result indicating low precision in simultaneously estimating these parameters. Putting constraints on some of the parameters using prior knowledge—either from calibration or physical insight—can mitigate this effect. The common procedure^24^ to investigate this is to first invert (back) the parameter covariance matrix provided by the Cramér–Rao analysis or by an experiment, retrieving the FIM. Then, add priors to the FIM and invert again to obtain an updated covariance matrix. The updated CRLB, in our formalism, will then be the square root of the diagonal elements of this matrix. If the prior is found to produce a strong decrease in the CRLB of the target parameter, we would typically try to establish a measurement that can produce the constraint correspond to the prior, for example, fixing the focus position or go to great lengths to carefully calibrate the roll-off parameter r. For a single parameter, this analysis procedure is captured in Eq. (10) for the focal position. If more parameters are controlled, or calibrated, it can be repeatedly applied, even when applied to the target parameter itself.^24^ Equation (10) also reveals that the highest gain in precision can be obtained by restricting a parameter, which has large initial covariance with the target parameter. For the attenuation coefficient, this will in general be the focal position or the Rayleigh length. Increasing precision of a parameter x, e.g., $p_{x}\to 0$ in Eq. (10), corresponds to adding an ever larger number to the corresponding element in the FIM. Rather than risk numerical instability in the Cramér–Rao analysis due to this, it should be considered to remove the rows and columns corresponding to the parameter from the FIM altogether so the parameter does not appear in the model as a variable anymore. In fact, we used this approach to reduce the Fisher matrix to a 2×2 matrix leading to the analytical expression of Eq. (9).

Our analysis can be extended to the depth resolved estimation (DRE) method for the attenuation coefficient, which has recently emerged as an alternative to the curve-fitting method.^27^ Following the same theoretical framework as described in Sec. 2, the CRLB for the estimated attenuation coefficient in the absence of shot noise is obtained as

σμOCT=μ^OCTM·N. (14)

The scaling with the number of averages and data points is identical to the curve-fitting method. However, contrary to the CRLB for curve fitting, which does not depend on the attenuation coefficient itself, the precision now depends on the estimated μ^OCT. The precision as well as the accuracy of the DRE method will be subject of a future publication.

## 5.1 Clinical Implication

Various studies have demonstrated the potential for tissue discrimination based on the attenuation coefficient. Patient studies can be undertaken to determine cut-off values from ROC analysis^9^^,^^28^ to discriminate between benign and malignant lesions. When an attenuation coefficient close to the cut-off value is found in a subsequent measurement, the relevance of that result in clinical decision making hinges on the precision of the individual OCT measurement. The CRLB analysis as presented in this article provides a convenient method to assess the precision as influenced by noise, by system properties such as the confocal parameters and by data processing steps such as the number of averages and AFR selection. Moreover, the analytical expression Eq. (9), which is valid when noise is negligible in the AFR (which is usually satisfied) and when the confocal parameters are well-known, directly gives the precision that can be obtained as a function of postprocessing parameters only.

In this article, we used parameter values that are typically encountered in OCT measurements in dermatology or urology. A Cramér–Rao analysis for ophthalmic applications was recently given by Ghafaryasl et al.,^23^ albeit with some procedural mistakes. The result of our Cramér–Rao analysis using the system parameters in Ref. 23 can be found in Appendix D. Promising clinical applications include spectroscopic OCT,^28^ where the attenuation coefficient is measured at multiple wavelengths to determine, e.g., oxygen saturation^29^ and hemoglobin concentration.^30^ Endeavors to measure water content^31^ and glucose levels^32^^–^^34^ based on the attenuation coefficient have also been undertaken. The precision with which these physiological properties can be determined, and the corresponding diagnostic relevance of such approaches squarely depends on the precision of the attenuation coefficient measurement.

## 6 Conclusion

In this article, we have used the Fisher information matrix to compute the maximum achievable precision of OCT attenuation coefficient determination. We specified this CRLB $\sigma_{\mu_{\mathrm{OCT}}}$ in the same units as the attenuation coefficient, e.g., ${\mathrm{mm}}^{-1}$, and derived an analytical expression for this bound in the absence of noise and assuming exact knowledge of OCT (confocal) system parameters. We have validated the calculations using numerical simulations. Results from the analytical expression are in good agreement with the standard deviation extracted from simulations when the SNR in the AFR is above 20 dB, which is usually obtainable in OCT measurements. Furthermore, our theoretical framework can be expanded to include the roll-off or be used to calculate the CRLB for the confocal parameters. Therefore, we strongly believe that this framework is an important advance toward the standardized clinical use of OCT-based tissue characterization and given its wide applicability, and we believe that our theoretical framework gives a valuable insight toward improvement and design of OCT measurements.

## 7 Appendix A: Analytical Expression for the Cramér–Rao Lower Bound for the Attenuation Coefficient

It is assumed that the focal position $z_{f}$ and Rayleigh length $z_{R}$ are known. In the absence of shot noise, this leads to a 2×2 Fisher matrix with $\theta =[\alpha ,\mu_{\mathrm{OCT}}]$ as the parameter array of interest. Starting from Eq. (8), the Fisher matrix simplifies to

$$F_{\theta }=\overset{M}{\underset{i=1}{\sum }}\frac{N}{\sigma_{A}^{2}(z_{i})}(\begin{array}{cc} \alpha^{-1} & -z_{i}\alpha^{-1/2}\\ -z_{i}\alpha^{-1/2} & z_{i}^{2} \end{array})\alpha T(z_{i}-z_{f})e^{-2\mu_{\mathrm{OCT}}z_{i}}.$$ (15)

For the normal distribution that is obtained from averaging Rayleigh random variables, we have the fixed relation between variance and mean $\sigma_{A}^{2}(z_{i})=\frac{c_{R}}{4}A^{2}(z_{i})$ so

$$F_{\theta }=\overset{M}{\underset{i=1}{\sum }}\frac{4N}{c_{R}}(\begin{array}{cc} \alpha^{-1} & -z_{i}\alpha^{-1/2}\\ -z_{i}\alpha^{-1/2} & z_{i}^{2} \end{array}).$$ (16)

An analytical solution for the CRLB for $\mu_{\mathrm{OCT}}$ can be obtained by replacing the sum by a definite integral.

For an AFR containing M independent data points, $\overset{M}{\underset{i=1}{\sum }}(\ldots )\to \frac{1}{\delta z}\int_{{\mathrm{AFR}}_{\min}}^{{\mathrm{AFR}}_{\max}}(\ldots )dz$, where δz is the pixel increment in the OCT data.

This leads to the 2×2 FIM

$$F_{\theta }=(\begin{array}{cc} \frac{4N({\mathrm{AFR}}_{\max}-{\mathrm{AFR}}_{\min})}{c_{R}\alpha \delta z} & -\frac{2N({\mathrm{AFR}}_{\max}-{\mathrm{AFR}}_{\min})({\mathrm{AFR}}_{\max}+{\mathrm{AFR}}_{\min})}{c_{R}\alpha^{1/2}\delta z}\\ -\frac{2N({\mathrm{AFR}}_{\max}-{\mathrm{AFR}}_{\min})({\mathrm{AFR}}_{\max}+{\mathrm{AFR}}_{\min})}{c_{R}\alpha^{1/2}\delta z} & \frac{4N({\mathrm{AFR}}_{\max}^{3}-{\mathrm{AFR}}_{\min}^{3})}{3c_{R}\delta z} \end{array}).$$ (17)

The parameter covariance matrix can be calculated analytically by matrix inversion of Eq. (17). With the aid of the determinant $|F_{\theta }|=F_{\theta 11}F_{\theta 22}-F_{\theta 12}F_{\theta 21}$

$$|F_{\theta }|=\frac{1}{3}(\frac{4N^{2}}{c_{R}^{2}\alpha \delta z^{2}}){\left({\mathrm{AFR}}_{\max}-{\mathrm{AFR}}_{\min}\right)}^{4}.$$ (18)

The covariance matrix becomes after some algebraic manipulations

$$F_{\theta }^{-1}=\frac{c_{R}}{N\cdot M}(\begin{array}{cc} \frac{\alpha ({\mathrm{AFR}}_{\max}^{3}-{\mathrm{AFR}}_{\min}^{3})}{{\left({\mathrm{AFR}}_{\max}-{\mathrm{AFR}}_{\min}\right)}^{3}} & \frac{3\alpha^{1/2}({\mathrm{AFR}}_{\max}+{\mathrm{AFR}}_{\min})}{2{\left({\mathrm{AFR}}_{\max}-{\mathrm{AFR}}_{\min}\right)}^{2}}\\ \frac{3\alpha^{1/2}({\mathrm{AFR}}_{\max}+{\mathrm{AFR}}_{\min})}{2{\left({\mathrm{AFR}}_{\max}-{\mathrm{AFR}}_{\min}\right)}^{2}} & \frac{3}{{\left({\mathrm{AFR}}_{\max}-{\mathrm{AFR}}_{\min}\right)}^{2}} \end{array}).$$ (19)

The diagonal elements of the covariance matrix correspond to the lower bounds on variance of amplitude and attenuation coefficient, respectively. The CRLB, expressed as standard deviation, is then given as

$$\sigma_{\mu_{\mathrm{OCT}},\mathrm{an}}=\frac{1}{{\mathrm{AFR}}_{\max}-{\mathrm{AFR}}_{\min}}\sqrt{\frac{3c_{R}}{N\cdot M}}.$$ (20)

## 8 Appendix B

CRLBs for the precision of all model parameters and corresponding simulation results for different parameters fixed or free running in the fitting procedure are presented in Table 2. Parameter set $\theta =[\alpha =2500,\mu_{\mathrm{OCT}}=2\;{\mathrm{mm}}^{-1},z_{f}=0.3\;\mathrm{mm},z_{R}=0.2\;\mathrm{mm},\text{ and }\sigma_{\text{shot}}=7]$. Prior to fitting, N=100 independent A-scans are averaged. ${\mathrm{AFR}}_{\min}=328\;\mu m$, ${\mathrm{AFR}}_{\max}=656\;\mu m$, |AFR|=328  μm, and sample points: M=41. The noise background $\sigma_{\text{shot}}=7$ corresponds to ${\mathrm{SNR}}_{\mathrm{AFR}}=37\;\mathrm{dB}$. The CRLB marked with (b) is calculated using the analytical expression Eq. (9); other values are calculated numerically from Eq. (8). The simulation results are the standard deviation of $10^{4}$ parameter estimations (Table 2). The columns listing results for $\sigma_{\mu_{\mathrm{OCT}}}$ are the same as Table 1.

**Table 2:** CRLBs for the precision of all model parameters and corresponding simulation results for different parameters fixed or free running in the fitting procedure.

Free running (✓) or fixed (—) parameter				$\sigma_{A}$ (−)				$\sigma_{\mu_{\mathrm{OCT}}}$ (${\mathrm{mm}}^{-1}$)				$\sigma_{zf}$ (mm)				$\sigma_{zR}$ (mm)			
A	$\mu_{\mathrm{OCT}}$	$z_{f}$	$z_{R}$	$\sigma_{\mathrm{shot}}=0$		$\sigma_{\text{shot}}=7$		$\sigma_{\text{shot}}=0$		$\sigma_{\text{shot}}=7$		$\sigma_{\text{shot}}=0$		$\sigma_{\text{shot}}=7$		$\sigma_{\text{shot}}=0$		$\sigma_{\text{shot}}=7$	
2500	$2\;{\mathrm{mm}}^{-1}$	0.3 mm	0.2 mm	CRLB	sim	CRLB	sim	CRLB	sim	CRLB	sim	CRLB	sim	CRLB	sim	CRLB	sim	CRLB	sim
✓	✓	—	—	107	210	107	210	0.086^a^	0.088	0.086	0.087	—	—	—	—	—	—	—	—
✓	✓	✓	—	216	140^b^	217	140^b^	0.34	0.46^b^	0.34	0.45^b^	0.1	0.02^b^	0.1	0.02	—	—	—	—
✓	✓	—	✓	790	1000^b^	790	1000^b^	0.89	0.67^b^	0.89	0.67^b^	—	—	—	—	0.13	0.08^b^	0.13^b^	0.13^b^
✓	✓	✓	✓	2000	9400^b^	2000	9400^b^	2.5	1.8^b^	2.5	1.7^b^	0.2	0.2	0.2	0.2	0.3	0.1^b^	0.3	0.1^b^

a Calculated from Eq. (9).

b Fitted parameter values were not normally distributed.

## 9 Appendix C

Upon averaging a number N of statistically independent A-scans, the distribution of the OCT amplitude values changes away from the Rayleigh distribution. Whereas the mean amplitude does not change; the variance is reduced by a factor N: $\sigma_{\left\langle A\right\rangle }^{2}=\frac{\sigma_{A}^{2}}{N}=\frac{c_{R}{\left\langle A\right\rangle }^{2}}{4N}$. When N≳30, the amplitudes in the averaged signal follow a Gaussian distribution with these means and variances.

**Fig. 5:**
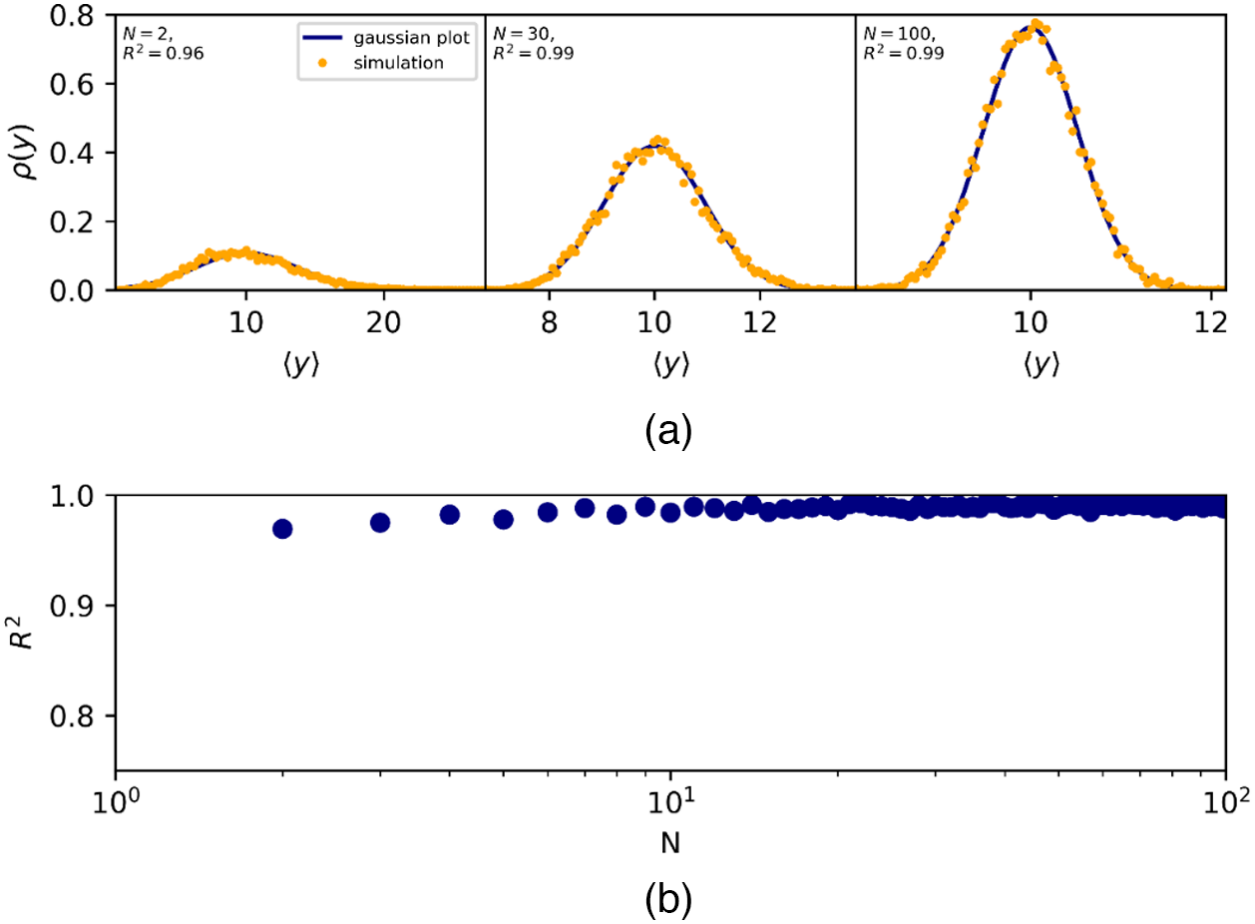
N-times averaged Rayleigh distributed random values ⟨y⟩ (orange dots, mean ⟨y⟩=10) compared to a Gaussian distribution (blue line) with the same mean and variance. (a) The probability distributions of ⟨y⟩ for N=[2,30,  100]. (b) The coefficient of determination, $R^{2}$ between the averaged Rayleigh and normal distributions as function of N (note the logarithmic horizontal scale). $R^{2}$ rises asymptotically towards 1, indicating that, for N≳30, the averaged Rayleigh variable can be approximated as being normally distributed.

To justify this estimation, we have numerically generated sets of $10^{4}$ Rayleigh distributed random values y parameterized by ⟨y⟩=10, and progressively averaged N=2−100 of these sets. For each value of N, a normal distribution was generated with mean ⟨y⟩ and variance $\sigma_{y}^{2}=\frac{c_{R}}{4N}{\left\langle y\right\rangle }^{2}$ (shown in Fig. 5(a) for N=[2,10,100]). We then calculated the coefficient of determination $R^{2}$ between the averaged dataset, and the generated normal distribution [shown in Fig. 5(b)] and find that $R^{2}$ asymptotically approaches 1 as N increases above 30.

## 10 Appendix D

**Fig. 6:**
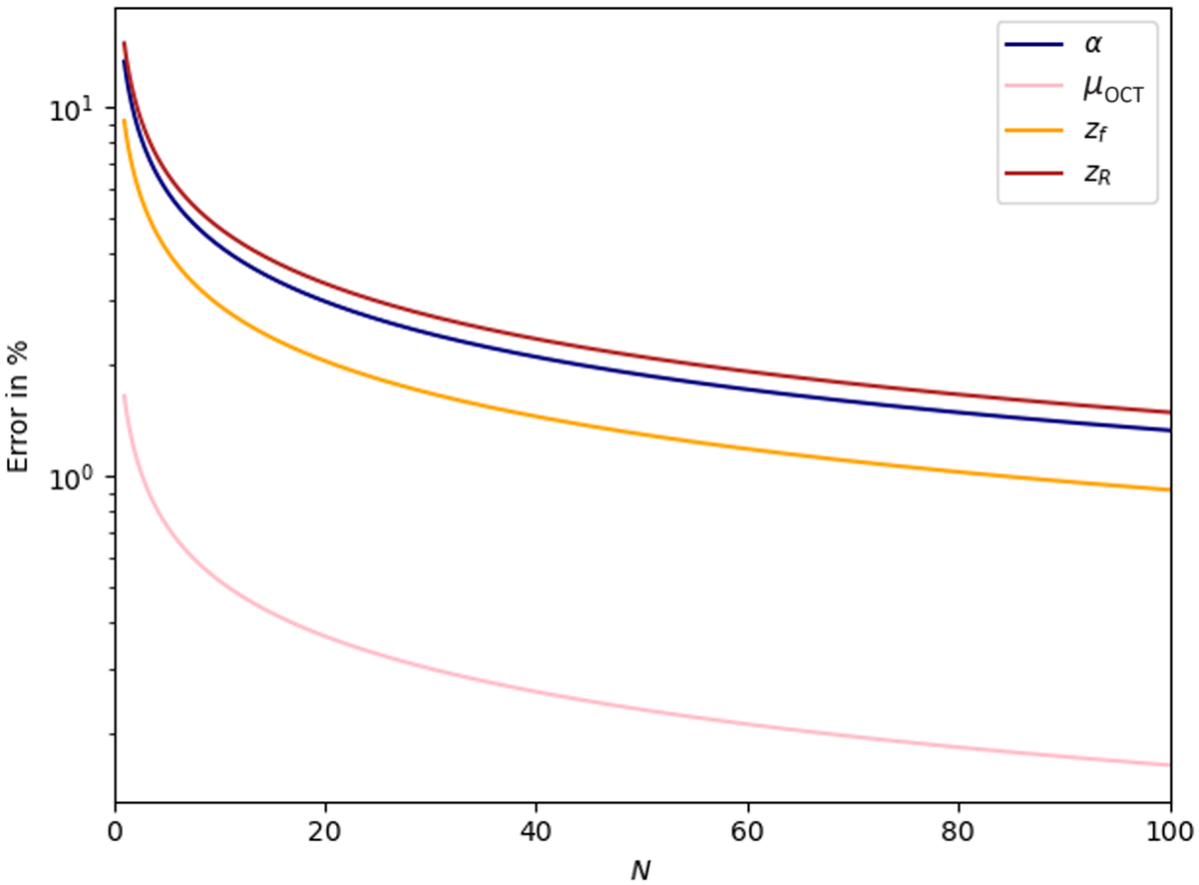
Relative CRLB on the precision of $\alpha ,\mu_{\mathrm{OCT}}$, $z_{f}$, and $z_{R}$ (lines) as a function of the number of averaged A-scans N. The Cramér–Rao bounds are calculated based on Eq. (8). Model parameters $\theta =[\alpha =25000,\mu_{\mathrm{OCT}}=0.72\;{\mathrm{mm}}^{-1},z_{f}=160\;\mu m,\text{ and }z_{R}=42\;\mu m]$. AFR=6.29  mm with M=1000 data points. *A priori* correction for noise is assumed.

**Fig. 7:**
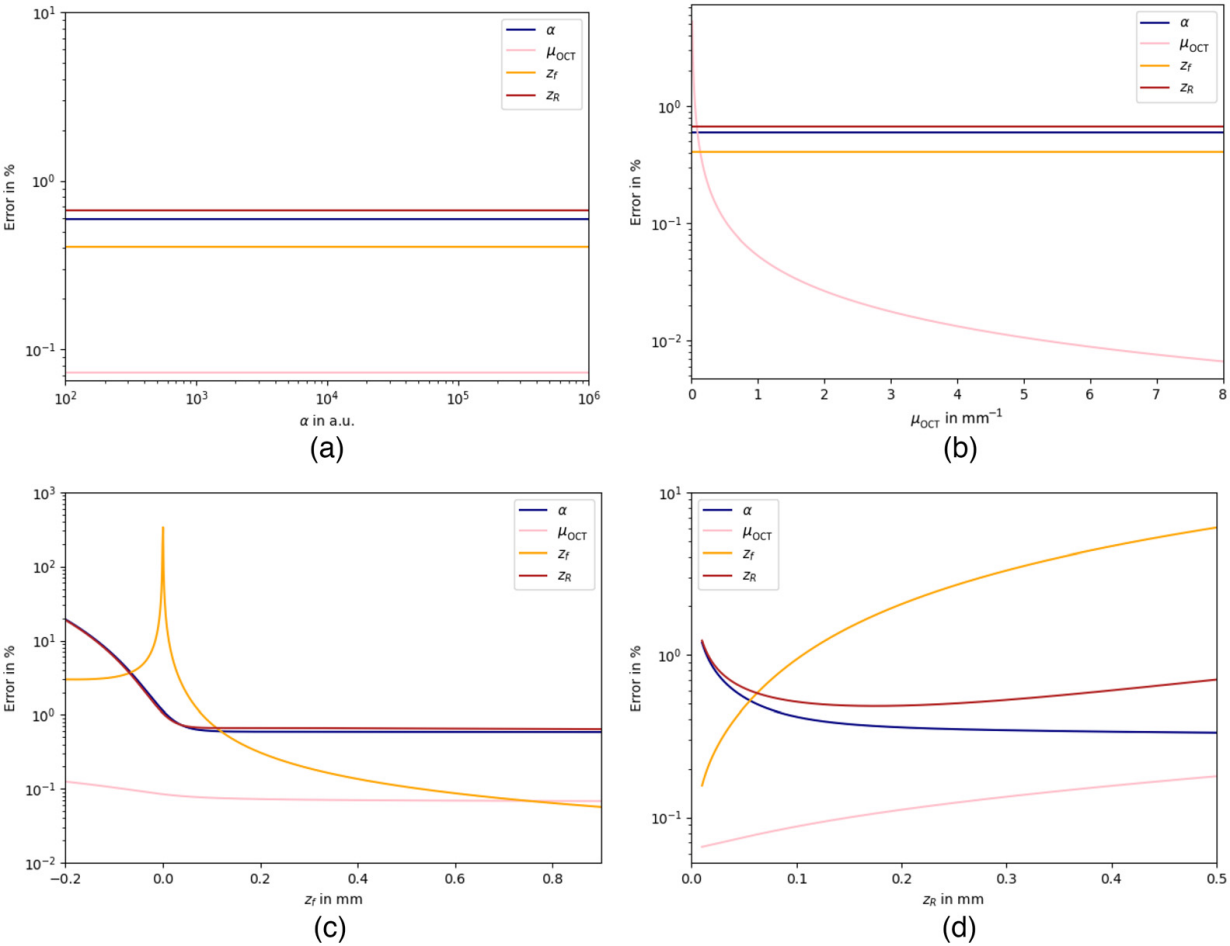
Relative CRLB on the precision of $\alpha ,\mu_{\mathrm{OCT}}$, $z_{f}$, and $z_{R}$ (lines) as a function of (a) α, (b) $\mu_{\mathrm{OCT}}\;$, (c) $z_{f}$, and (d) $z_{R}$. Other parameters are fixed at their base values from the set $\theta =[\alpha =25000,\mu_{\mathrm{OCT}}=0.72\;{\mathrm{mm}}^{-1},z_{f}=160\;\mu m,\text{ and }z_{R}=42\;\mu m]$. The CRLBs are calculated based on Eq. (8). AFR=6.29  mm with M=1000 data points. *A priori* correction for noise is assumed.

A Cramér–Rao analysis for the parameter set $\theta =[\alpha =25000,\mu_{\mathrm{OCT}}=0.72\;{\mathrm{mm}}^{-1},z_{f}=160\;\mu m,\text{ and }z_{R}=42\;\mu m]$ representative for ophthalmic OCT systems was previously reported in Ref. 23, using the same signal model as in the present manuscript. Before the analysis, the signal is already noise corrected. Instead of CRLBs expressed as standard deviations, the relative CRLB were reported (e.g., normalized on the parameter values). For comparison, we calculated accordingly the relative lower bounds using Eqs. (7) and (8) while setting the noise floor to zero (ζ=0, $\sigma_{\text{shot}}=0$). Similar to Ref. 23, N=500 A-scans were taken for averaging, yet a more realistic axial increment of $\delta_{z}=8\;\mu m$ was used instead of 1.27  μm. The AFR was 6.29 mm. The rCRLB on the parameters α, $\mu_{\mathrm{OCT}}$, $z_{f}$, and $z_{R}$ as a function of the number of A-scans taken for averaging N=[0,1,…,99,100] is shown in Fig. 6, resulting in an expected $1/\sqrt{N}$-dependency for all four parameters. In addition, Figs. 7(a)–7(d) show the rCRLB for the four parameters versus the value of the parameter $\alpha =[10^{2}-10^{6}]$ in a.u., $\mu_{\mathrm{OCT}}=[0.01-8]$ in ${\mathrm{mm}}^{-1}$, $z_{f}=[-0.2-0.9]$ in mm, and $z_{R}=[0.01-0.5]$ in mm while using 1000 equally distributed data points. Please note that our numerical evaluations, which are shown in Figs. 6 and 7, result in a different relative error as reported in Ref. 23 due to some procedural mistakes in Ref. 23. In OCT, the variance is directly related to the mean value [Eq. (5)] and is thus variable for each data point. However, all relative errors are below the 10% threshold as stated in Ref. 23 upholding their main conclusion.

## Code, Data, and Materials Availability

The code is available from the authors upon request.
